# Supplementary material for: Synthesis, Characterization, DFT and Photocatalytic Studies of a New Pyrazine Cadmium(II) Tetrakis(4-methoxy-phenyl)-porphyrin Compound
Source: Molecules. 2022 Jun 14;27(12):3833. doi: 10.3390/molecules27123833 (PMC9227090; doi:10.3390/molecules27123833)
Supplement: Supplementary file 1 [file molecules-27-03833-s001.zip › molecules-1718867-Supplementary Materials.pdf]

Supplementary Materials

Table S1. Crystallographic data of [Cd(TMPP)Pyz] (**1**).

Formula	$C_{52}H_{40}CdN_6O_4$
Formula weight	925.30
Temperature (K)	150
Space group	P21/c
Crystal system	monoclinic
a/ Å	23.0965(10)
b/ Å	17.6690(7)
c/ Å	12.4340(5)
α /(deg)	90
β /(deg)	99.551(2)
γ /(deg)	90
Volume/Å3	5003.9(4)
Z	4
Density (g/ cm-3)	1.228
Crystal size (mm3)	0.360 × 0.290 × 0.250
Absorption coefficient (mm-1)	0.484
F(000)	1896.0
Reflections collected	37209
Independent reflections	11304[R(int) = 0.0284]
Data/restraints/parameters	11304/0/572
Index ranges (h. k. l)	-28. 29; -22. 22; -13. 16
Theta range for data collection θ (°)	5.836 to 54.92°
S [Goodness of fit]	1.030
R1 a[Fo>4σ(Fo)]	0.0355
wR2b ([all data])	0.0879
Largest diff. peak/hole / e Å-3	0.60/-0.59
CCDC	2069722

[a]: R1 =Σ||Fo|–|Fc||/Σ|Fo|.b: wR2 ={Σ[w(|Fo|2–|Fc|2)2]/Σ[w(|Fo|2)2]}1/2.

Table S2. Selected bond lengths (Å) and dihedral angles (°) for [Cd(TMPP)(Pyz)] (**1**).

Cadmium coordination polyhedron		
Bond lengths (Å)	Experimental	Theoretical
Cd-N1	2.1742(18)	2.22
Cd-N2	2.1813(17)	2.23
Cd-N3	2.1949(17)	2.23
Cd-N4	2.2010(17)	2.22
Cd-N5	2.3696(19)	2.46
Angles (°)	Experimental	Theoretical

N1-Cd-N2	86.61(6)	85.56
N1-Cd-N3	149.41(7)	143.91
N1-Cd-N4	86.37(6)	84.78
N1-Cd-N5	115.70(7)	124.60
N2-Cd-N3	86.22(6)	84.06
Pyrazine (axial ligand)		
Bond lengths (Å)	Experimental	Theoretical
N5-C62	1.338(3)	1.34
N5-C66	1.332(3)	1.34
C62-C63	1.372(4)	1.39
N6-C63	1.336(4)	1.34
N6-C65	1.323(4)	1.34
Angles (°)	Experimental	Theoretical
N5-C66-C65	121.4(2)	122.08
N5-C62-C63	121.6(2)	122.09
C62-N5-C66	116.3(2)	115.83
C63-N6-C65	115.5(2)	115.83
N6-C63-C62	122.4(3)	122.08
N6-C65-C66	122.9(3)	122.09

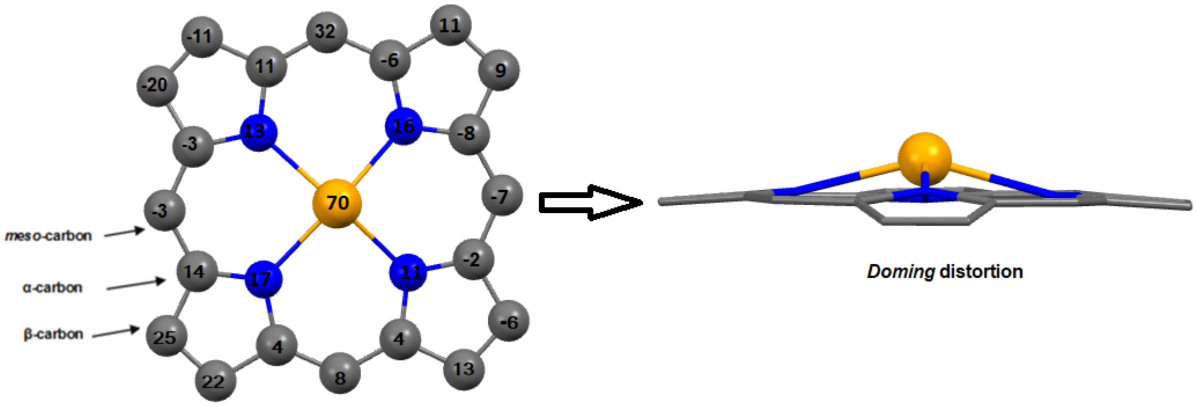

Figure S1. Formal diagrams of the porphyrinato core showing the out-of-plane localization of Cd(II) metal ion in [Cd(TMPP)(Pyz)] (**1**).

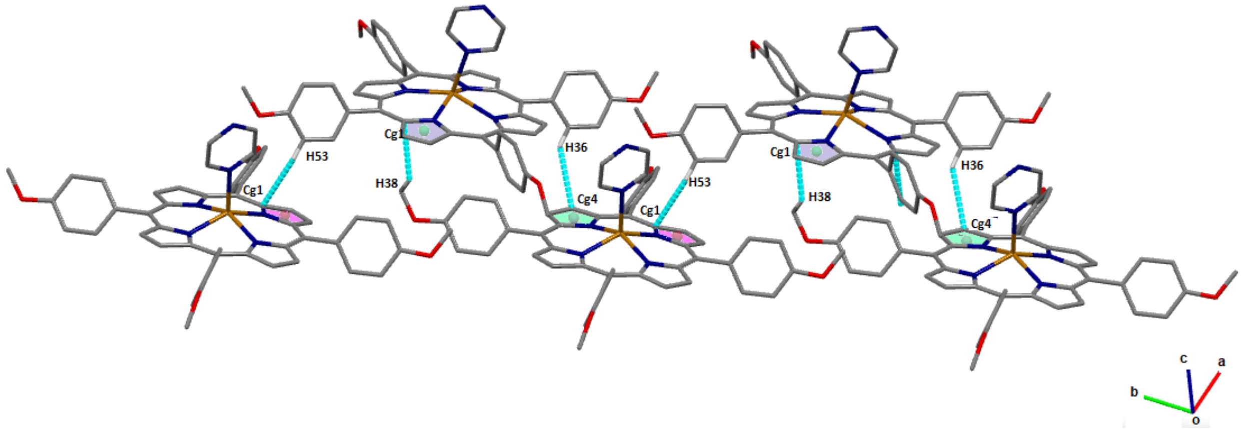

Figure S2. Schematic representation showing the C-H···Cg intermolecular interactions contacts are shown as dashed lines.

Table S3. Selected intermolecular C-H···N and C-H···π interactions for [Cd(TMPP)(Pyz)] (**1**).

D-H···A a	Symmetry of A	D-H···A (Å)	D-H···A (°)
C48-H48···N6	1+x,-1+y, z	2.676(2)	148
C65-H65···N6	1-x, 1+y, z	2.962(3)	143
C38-H38···Cg1b	X,1/2-Y,-1/2+Z	3.661(3)	158
C53-H53···Cg4c	X,3/2-Y,1/2+Z	3.639(4)	148

[a]: D is the donor atom and A is the acceptor atom, b: Cg1 is a pyrrole centroid (*i.e.* C20-C19-C18-C1-N1), and c: Cg4 is a pyrrole centroid (i.e. C16-C15-C14-C13-N4).

Table S4. Total interaction energies for [Cd(TMPP)(Pyz)] (**1**) (KJ.mol^{-1}) at the B3LYP/DGDZVP basis set

	N	Sym op	R	E_{ele}	E_{pol}	E_{dis}	E_{rep}	E_{tot}
	2	x, y, z	17.67	7.6	-1.3	-24.8	0.0	-14.5
	2	x, -y+1/2, z+1/2	12.01	-15.8	-5.0	-71.4	0.0	-82.6
	2	x, -y+1/2, z+1/2	9.66	-47.9	-8.1	-123.1	103.1	-100.2
	2	x, y, z	12.43	-2.3	-0.9	-12.0	0.0	-13.6
	2	-x, y+1/2, -z+1/2	16.94	-3.9	-1.1	-16.0	0.0	-18.8
	1	-x, -y, -z	18.03	-2.7	-1.4	-25.9	0.0	-26.4

	1	-x, -y, -z	10.55	-40.5	-8.1	-100.2	84.3	-83.9
	2	-x, y+1/2, -z+1/2	16.59	-2.6	-1.3	-24.7	0.0	-25.2
	2	-x, y+1/2, -z+1/2	13.28	-3.5	-1.1	-11.7	0.0	-14.7
	1	-x, -y, -z	12.92	-7.1	-1.0	-9.5	0.0	-16.6

Table S5. Total interaction energies for H_2-TMPP (KJ.mol^{-1}) at the B3LYP/DGDZVP basis set.

	N	Sym op	R (Å)	E_{ele}	E_{pol}	E_{dis}	E_{rep}	E_{tot}
	2	-x, y, -z+1/2	9.83	-20.8	-3.6	-62.4	47.7	-49.5
	4	-x, y, -z+1/2	16.41	-0.3	-1.0	-18.1	0.0	-16.7
	2	x, y, z	17.07	0.8	-0.2	-4.9	0.0	-3.6
	2	x, y, z	13.93	-15.3	-2.5	-40.8	0.0	-53.6
	2	-x, y, -z+1/2	8.68	-56.7	-8.5	-130.1	176.7	-70.4
	2	x, y, z	15.70	-3.9	-3.1	-46.2	0.0	-46.6

Table S6. Atomic percentage contribution to the distance between the surface and the internal and external nucleus to the surface for H_2-TMPP and [Cd(TMPP)(Pyz)] (**1**).

% Contribution											
H_2-TMPP											
Inside-Outside						Inside-Outside					
C-H	C-N	C-O	O-H	N-H	N-O	H-C	N-C	O-C	H-O	H-N	O-N
12.8	0	0.4	5.4	2.7	0	11.0	0	0.3	4.9	2.2	0
% Contribution											
[Cd(TMPP)(Pyz)] (**1**)											
Inside-Outside						Inside-Outside					
C-H	C-N	C-O	N-O	N-H	O-H	H-C	N-C	O-C	O-N	H-N	H-O
13.6	0	0.5	0.1	4.3	4.9	11.5	0	0.4	0.1	3.3	4.3

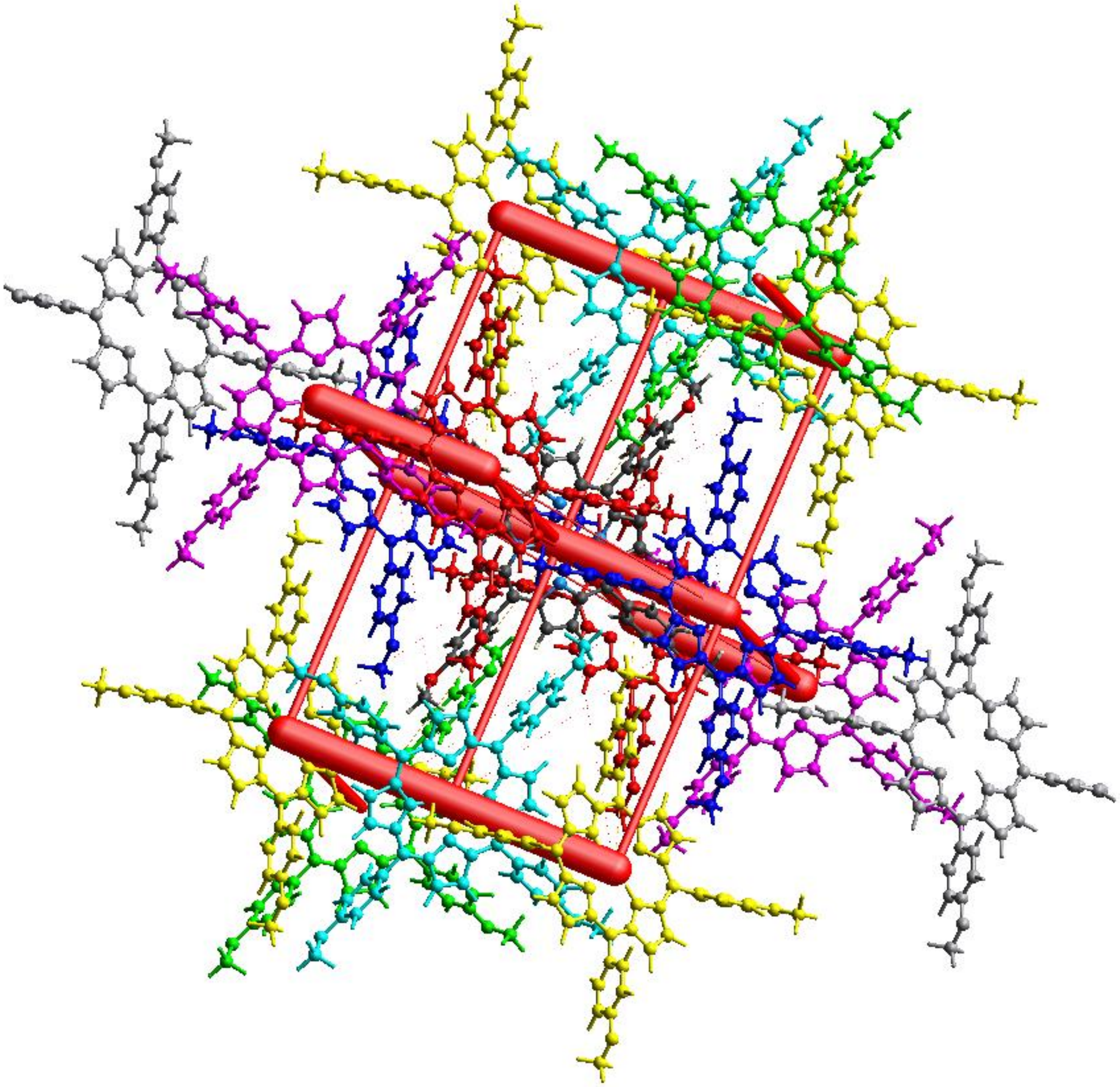

Figure S3. Coulombic interacting energies for interacting molecules in the H_2-TMPP crystal.

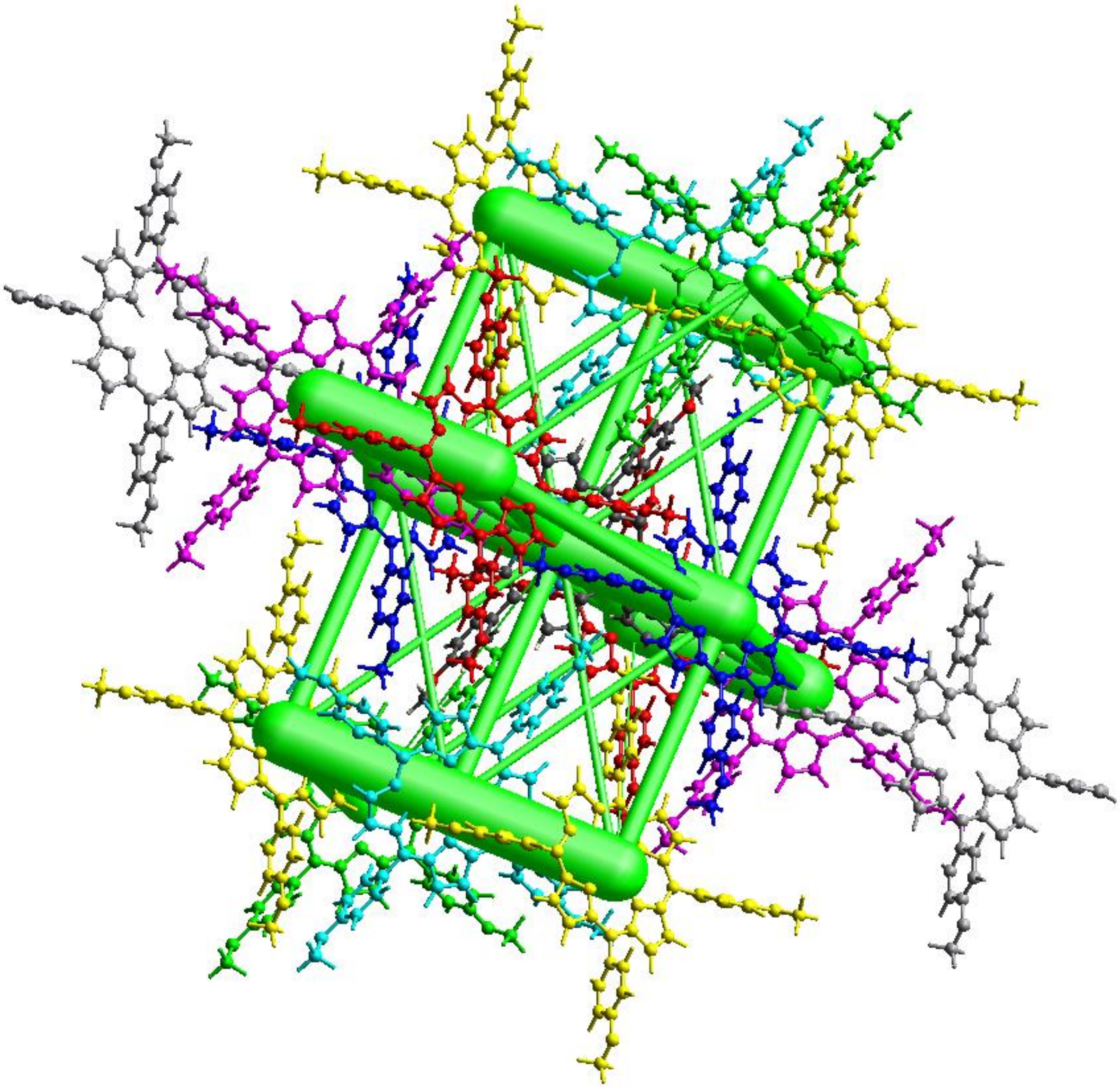

Figure S4. Dispersion interacting energies between molecules in the H_2-TMPP crystal.

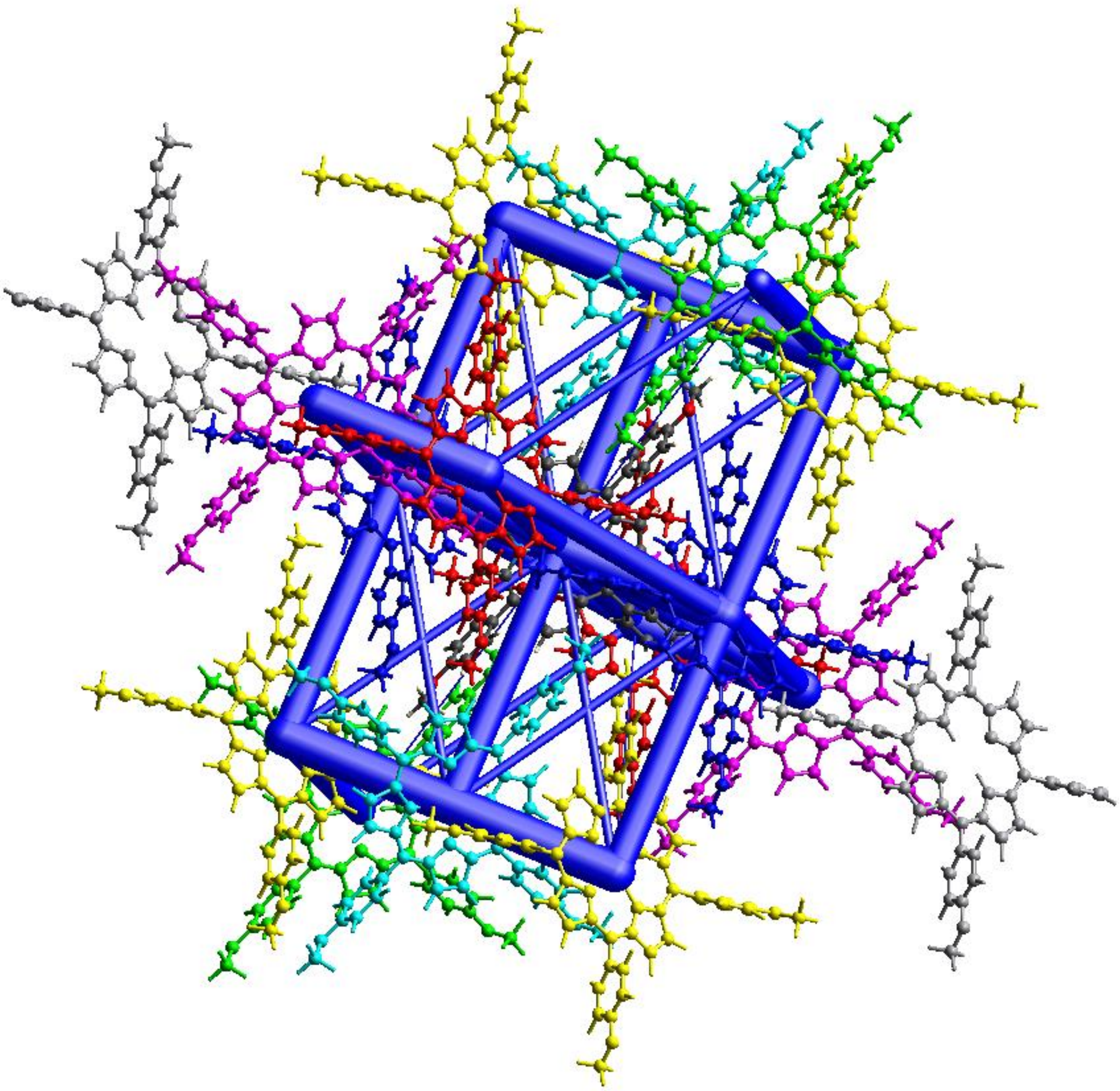

Figure S5. Total interacting energies between molecules in the H_2-TMPP crystal.

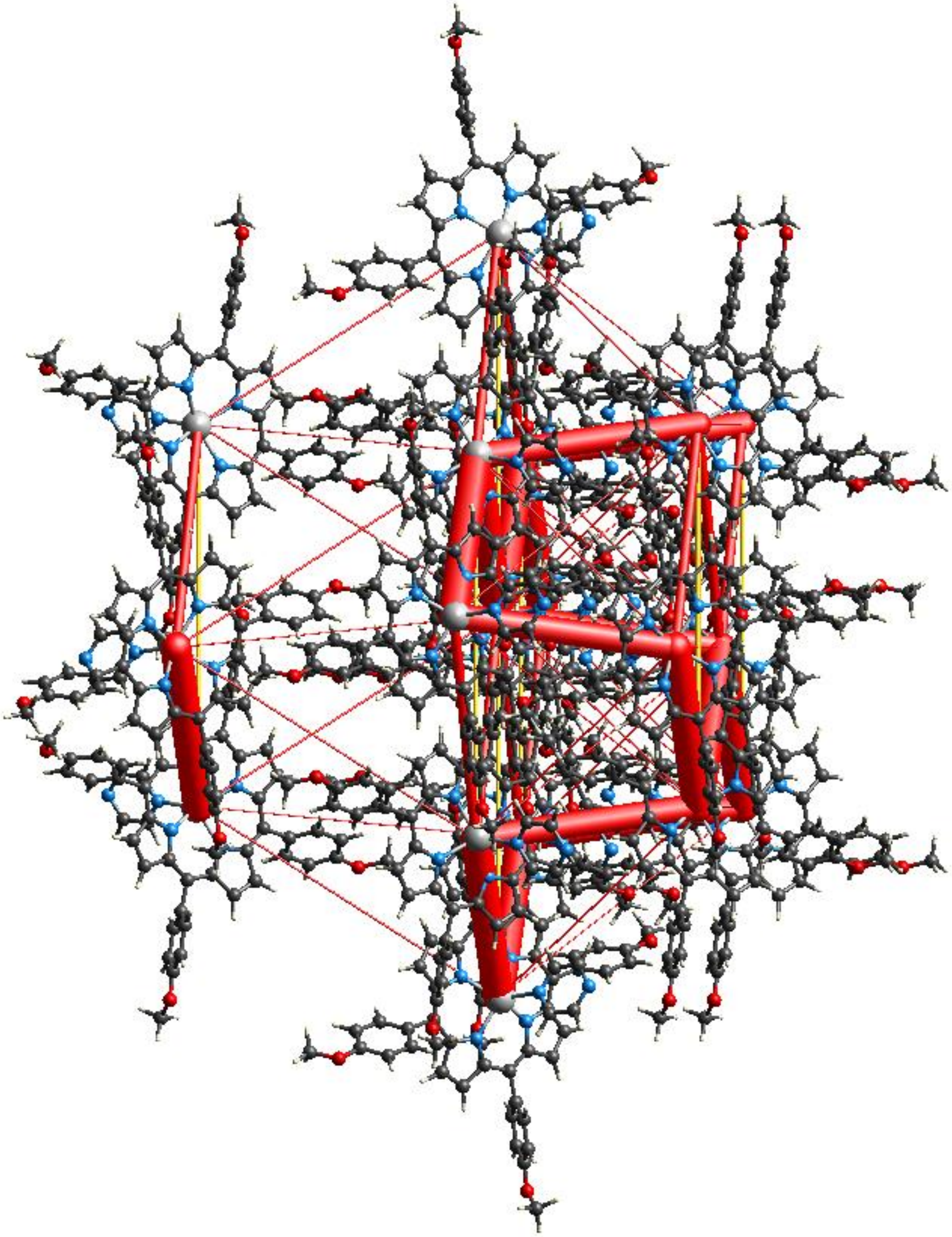

Figure S6. Coulomb interacting energies between molecules in the [Cd(TMPP)(Pyz)] crystal.

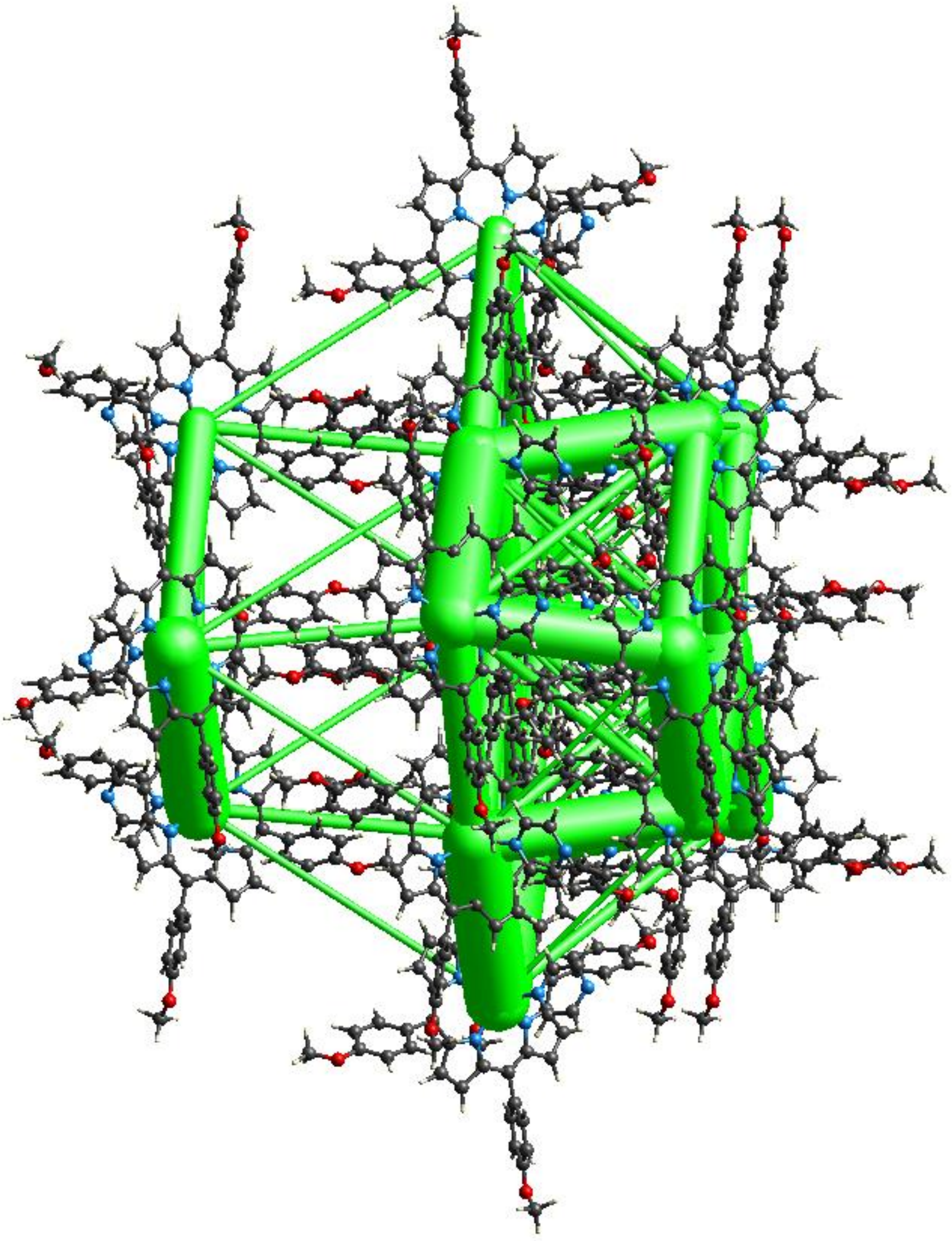

Figure S7. Dispersion interacting energies between molecules in the [Cd(TMPP)(Pyz)] crystal.

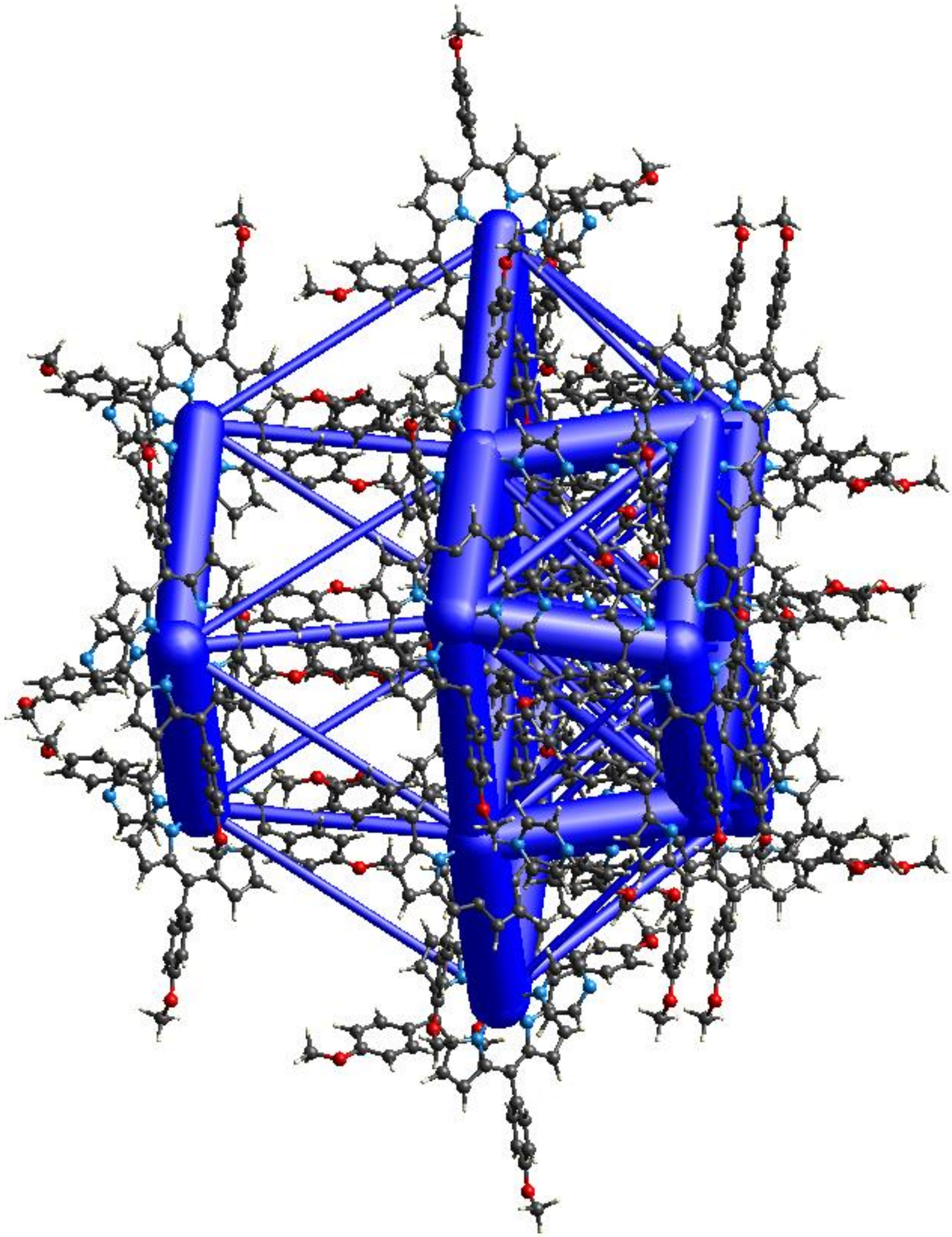

Figure S8. Total interacting energies between molecules in the [Cd(TMPP)(Pyz)] crystal.

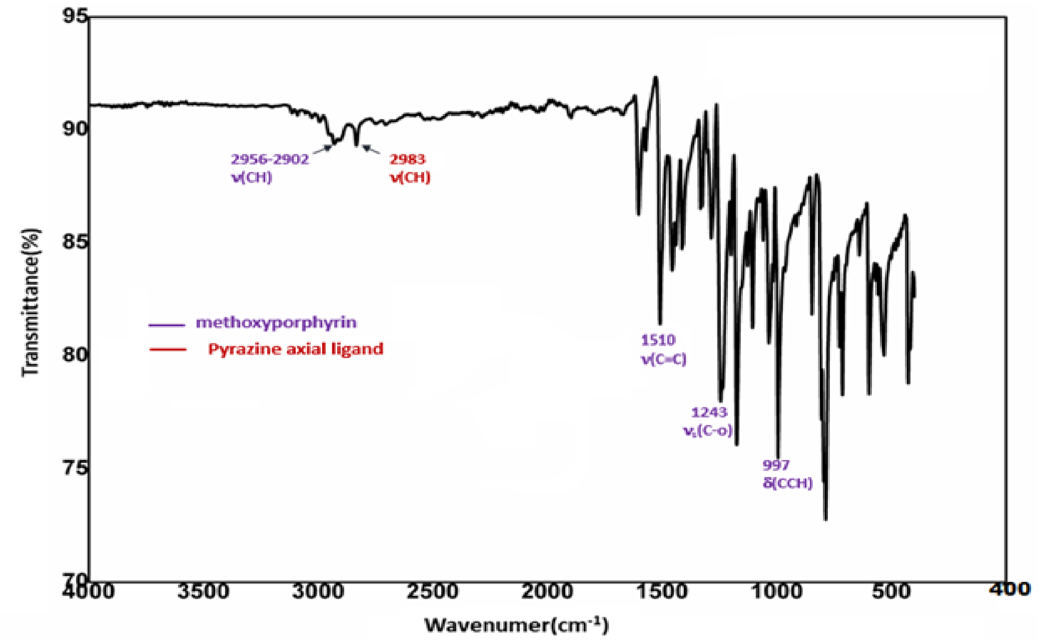

Figure S9. FTIR spectrum of solid [Cd(TMPP)(Pyz)] (**1**).

Table S7. Experimental and Theoretical IR assignments of the complex (**1**).

	[Cd (TMPP)(Pyz)] **(1)**	
Experimental (cm^{-1})	Theoretical (cm^{-1})	Assignment
1243	1290	(O–CH3)
1510	1561	(C=C)
2833	3203	(C-H)Pyz
2956-2902	3215-3282	(C-H)proph
997	1009	((δ(CCH))porph

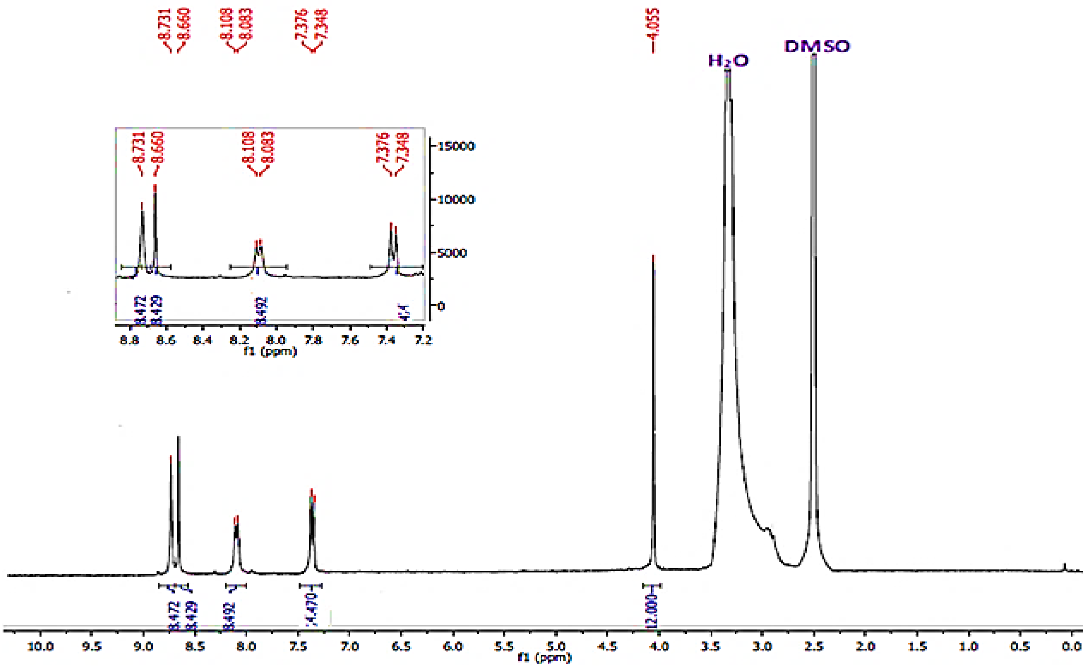

Figure S10. ^{1}H NMR spectrum (300 MH z, DMSO-d6, 298 K) of [Cd (TMPP)(Pyz)] (**1**).

Table S8. ^{1}H NMR data (δ in ppm) of the [Cd(TMPP)(Pyz)] complex in DMSO-*d*$_6$.

H (ppm)	Experimental	Theoretical
**Hβ-pyr**	8.73	8.1
**Ho-Ph/ Hm-Ph**	8.10/ 8.08	6.1
**p- OCH$_3$**	4.05	3.9
**H(Pyz ligand)**	7.34	8.2-8.7

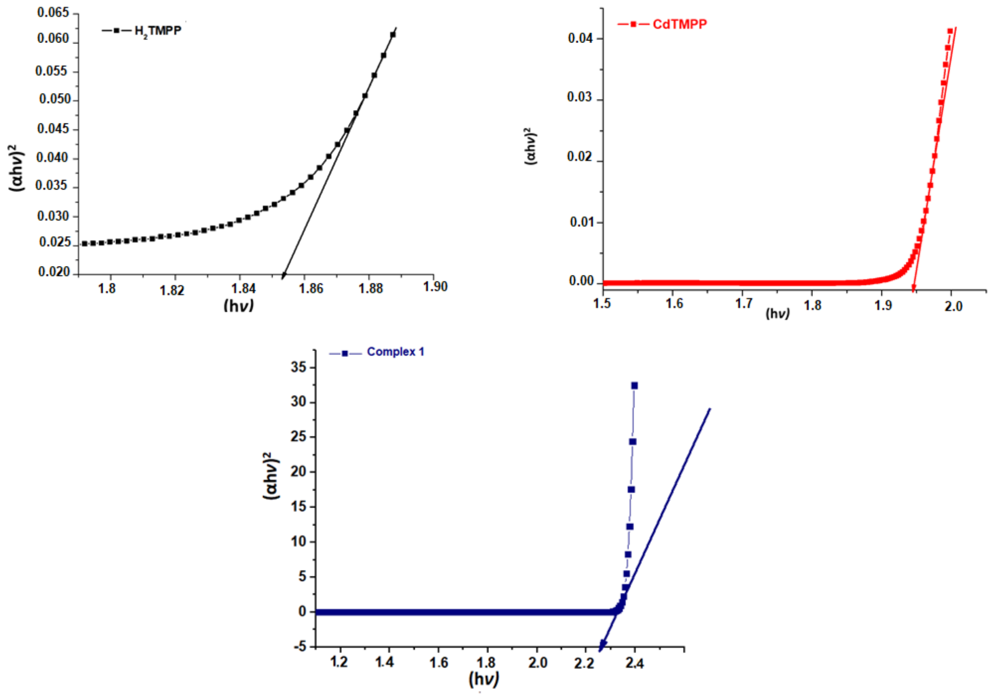

Figure S11. Plots of $(\alpha h\upsilon)^2$ vs photon energy E of H_2TMPP, CdTMPP and Complex **1**. α is the absorption coefficient.

Table S9. UV-Visible data of H_2TMPP, [Cd(TMPP)], [Cd(TMPP)(Pyz)] and several [Cd(porph)(L)] porphyrin complexes .

Compound	Soret Band [a,b]	Q bands [a,b]	Eg (eV)	Ref.
H_2-TMPP[c]	422 (463.2)	518 (14.13), 556 (9.7), 593 (4.3), 650 (4.8)	-	[61]
[Cd(TMPP)] [c]	436(367.7)	572(15.02), 616(15.87)	-	[61]
H_2-TMPP[c]	422(390.2)	518(14.10), 555(9.5), 594(3.9), 650(4.2)	1.85	This work
[Cd(TMPP)][c]	437(125)	573(4.2), 617(2.4)	1.95	This work
H_2-TClPP[c]	420 (309)	517 (15.8), 553(9.2), 589 (5.6), 645 (4.1)	1.820	[36]
[Cd(TClPP)][c]	433 (117)	568 (2.3), 609(2.6)	1.960	[36]
[Cd(T(p-Cl)4PP)(py)][c]	429(301)	– –	–	[62]
[Cd(T(p-Cl)4PP)(DMF)][c]	433(310)	567(18), 609(14)	–	[62]
[Cd(TPP)(2-NH_2-py)][d]	433(310)	567(18), 609(14)	–	[25]
[Cd(TClPP)(morph)] c	436(126)	575(15.84), 620(3.6)	1.903	[36]
[Cd(TBPP)(2-MeHIm)][c]	438 (109.65)	579 (14.65), 621 (3.5)	1.920	[22]
[Cd(TMPP)(Pyz)] [c]	440(113)	579(15.2) 623(3.2)	2.23	This work

[a]: λ_{max} (nm), [b]: log ε. (ε. 10^{-3} L.cm^{-1}.mol^{-1}), [c]: in dichloromethane solvent, [d]: in chloroform solution.

Table S10. Photophysical data of our synthetic porphyrin species and a selection of Cd(porphyrin) complexes were recorded in CH_2Cl_2.

Compound	Fluorescence [a] λmax (nm)		Φf [b]	τf (ns) [c]	ΦΔ [d]	Ref.
	Q(0,1)	Q(0,0)				
H_2-TClPP[e]	653	678.8	0.063	8.2	0.69	[63]
H_2-TClPP	650	715	0.075	8.9	0.49	[36]
H_2-TBPP	650	717	0.08	8.9	-	[22]
H_2-TMPP	656	722	0,084	7.8	0.73	This work
[Cd(TPP)][f]	620	655	0.065	-	0.69	[33]
[Cd(TMPP)][g]	622	667	0.018	-	0.73	[61]
[Cd(TClPP)]	619	650	0.06	1.6	0.41	[36]
[Cd(TClPP)(morph)]	619	652	0.046	1.5	0.53	[36]
[Cd(TBPP)]	614	650	0.01	1.6	-	[22]
[Cd(TBPP)(2-MeIm)]	612	657	0.02	1.7	-	[22]
[Cd(TMPP)(DABCO)]	625	652	0.01	1.2	-	[21]
[Cd(TMPP)]	618	652	0.06	1.8	0.57	This work
[Cd(TMPP)(Pyz)](**1**)	618	652	0.03	1.6	0.13	This work

[a]: Fluorescence spectra of porphyrins were obtained as a function of λex = 437 nm. [b]: Fluorescence quantum yield. [c]: Fluorescence lifetime (ns). [d]: Singlet oxygen production quantum yield. [e]: in toluene solvent, [f]: in EtOH solvent, [g]: in CH_2Cl_2 solvent.

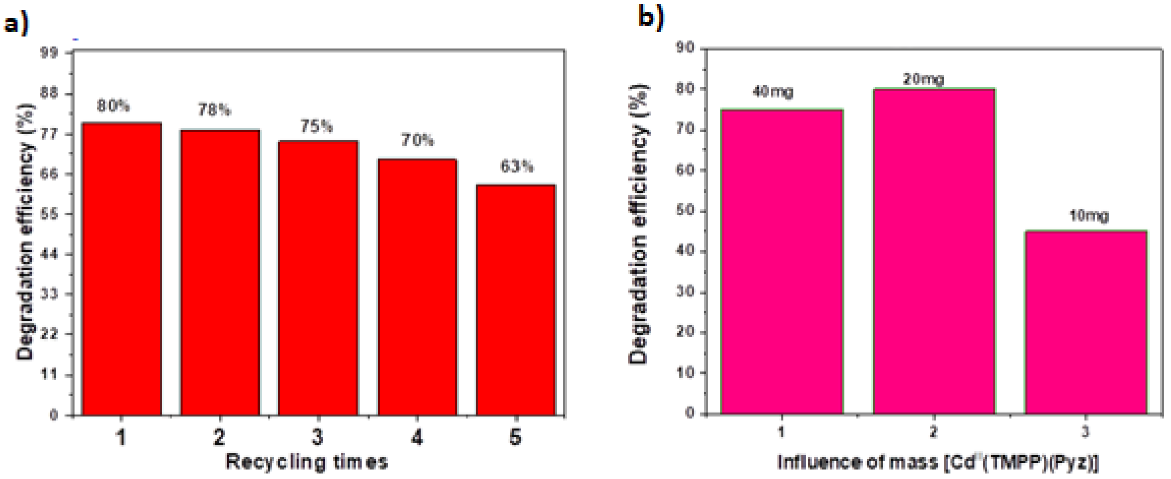

Figure S12. (**a**) Cycling experiments. (**b**) Influence of mass of [Cd^{II}(TMPP)(Pyz)] (**1**).
